# Non-Cardiac Comorbidities in Acute Heart Failure: Phenotype-Specific Insights from Sub-Saharan Africa

**DOI:** 10.3390/jcm15062202

**Published:** 2026-03-13

**Authors:** Umar G. Adamu, Samantha Nel, Confidence Makgoro, Muzi Maseko, Nqoba Tsabedze

**Affiliations:** 1 Division of Cardiology, Department of Internal Medicine, Faculty of Health Sciences, University of the Witwatersrand, Johannesburg 2193, South Africa; samantha.nel@wits.ac.za (S.N.); confidence.makgoro@wits.ac.za (C.M.); nqoba.tsabedze@wits.ac.za (N.T.); 2 Nutrition and Hypertension Laboratory, Department of Physiology, Faculty of Health Sciences, University of the Witwatersrand, Johannesburg 2193, South Africa; muzi.maseko@wits.ac.za

**Keywords:** heart failure, non-cardiac comorbidities, phenotypes, in-hospital mortality, sub-Saharan Africa, multimorbidity

## Abstract

**Background:** Non-cardiac comorbidities (NCCs) are highly prevalent among patients hospitalized for acute heart failure (HF). However, data from sub-Saharan Africa (SSA) on their distribution across HF phenotypes and association with in-hospital outcomes remain limited. **Methods:** We prospectively enrolled adults hospitalized with acute HF at a tertiary centre in South Africa between February and November 2023. Ten NCCs were assessed and patients were categorized according to comorbidity burden. The primary outcomes were all-cause in-hospital mortality and length of stay. Multivariable regression and sensitivity analyses were performed to identify predictors of outcomes. **Results:** Of the 406 patients (mean age 54.9 ± 15.8 years; 51% women), HF with reduced ejection fraction (HFrEF) accounted for 63%, HF with mildly reduced ejection fraction (HFmrEF) for 15%, and HF with preserved ejection fraction (HFpEF) for 21%. The most common NCCs were diabetes (47%), chronic kidney disease (CKD) (46%), obesity (45%), and anaemia (33%). Two-thirds had ≥2 NCCs. The median hospital stay was 8 days (IQR: 5–12) and in-hospital mortality was 3.4% (*p* > 0.05 across NCC groups). Higher heart rate predicted longer hospitalization, while renin angiotensin system inhibitor (RASi) therapy was associated with shorter stay. Lower Kansas City Cardiomyopathy Questionnaire (KCCQ) score (adjusted odds ratio [aOR] 1.009; 95% confidence interval [CI]: 1.003–1.015) and higher log-transformed NT-proBNP were independently associated with increased in-hospital mortality (aOR 1.85; 95% CI: 1.07–3.50; *p* = 0.026). Total comorbidity burden was not independently associated with length of stay or in-hospital mortality. **Conclusions:** Non-cardiac comorbidities are common in acute HF in SSA, and functional status and clinical markers were the strongest predictors of length of stay and in-hospital mortality.

## 1. Introduction

Heart failure (HF) is a major global health challenge, affecting approximately 64.3 million individuals worldwide and contributing substantially to hospitalization and premature death [1]. Acute HF, characterized by the rapid onset or worsening of symptoms, imposes a considerable healthcare burden, particularly in low- and middle-income countries, where resources are limited and access to optimal care is constrained [2,3].

Non-cardiac comorbidities (NCCs), including diabetes, chronic kidney disease (CKD), obesity, and anaemia, are increasingly recognized as key determinants of poor outcomes in patients hospitalized with acute HF [4,5,6]. These comorbidities exacerbate hemodynamic stress, impair therapeutic response, and complicate clinical management. Large registries from high-income countries, such as the ESC-HFA EURObservational Research Programme Heart Failure Long-Term Registry, report that up to 80% of patients hospitalized with acute HF have at least one NCC, which is associated with prolonged hospitalization, higher in-hospital mortality, and suboptimal use of guideline-directed medical therapy (GDMT) [5].

In contrast, evidence from sub-Saharan Africa (SSA) remains limited. The region faces a dual burden of communicable diseases, such as HIV, and non-communicable diseases, including hypertension and diabetes, which may uniquely shape comorbidity profiles and HF trajectories [7,8]. Patients in SSA often present with HF at a younger age [9,10,11]. Furthermore, the distribution of HF phenotypes, including HF with reduced ejection fraction (HFrEF), HF with mildly reduced ejection fraction (HFmrEF), and HF with preserved ejection fraction (HFpEF) may differ substantially from that observed in high-income countries [9,10,11].

Given these demographic and epidemiological distinctions, a focused evaluation of NCC prevalence and it clinical impact in SSA is needed to guide risk stratification, optimize resource allocation, and develop context-appropriate management strategies. Characterizing patterns of NCCs across HF phenotypes may further inform personalized therapeutic approaches. Therefore, this study aimed to determine the prevalence of NCCs and evaluate their association with in-hospital outcomes among adults hospitalized with acute HF in Johannesburg, South Africa.

## 2. Materials and Methods

### 2.1. Study Design and Population

**Figure 1:**
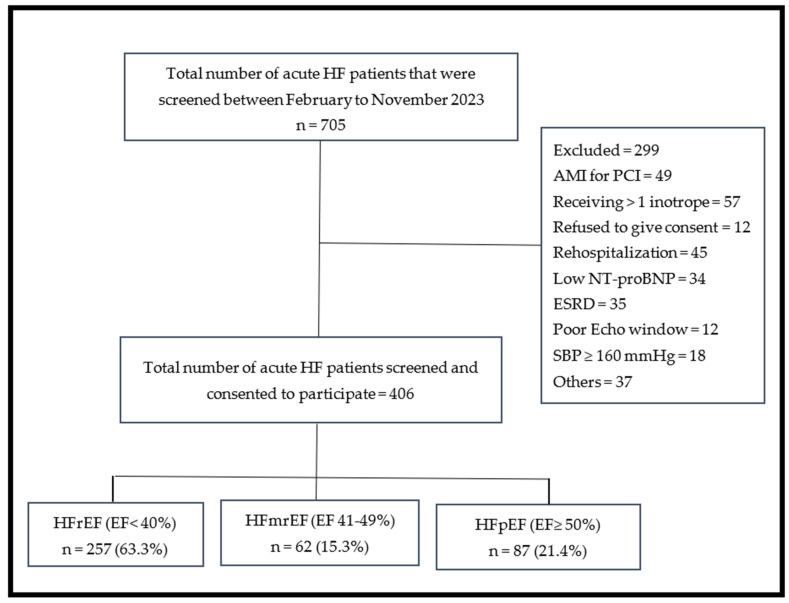
Flowchart showing the distribution of patients with acute HF by HFsubtypes. AMI = Acute myocardial infarction; Echo = Echocardiography; EF = Ejection fraction; ESRD = End-stage renal disease; HFmrEF = heart failure with mildly reduced ejection fraction; HFpEF = heart failure with preserved ejection fraction; HFrEF = heart failure with reduced ejection fraction; NT-proBNP = N-terminal pro-B-type natriuretic peptide; SBP = systolic blood pressure.

This was a cross-sectional observational study conducted at Charlotte Maxeke Johannesburg Academic Hospital (CMJAH), a 1088-bed state-owned tertiary referral healthcare facility in Johannesburg, South Africa, between February and November 2023. The baseline characteristics, cardiovascular risk factors, and the primary outcomes of the study have been published [11]. We enrolled 406 consecutive patients aged ≥18 years admitted with acute HF, as defined by the 2021 ESC guidelines [2]. Other inclusion criteria included New York Heart Association (NYHA) class II–IV symptoms and elevated N-terminal proB natriuretic peptide (NT-proBNP). We excluded patients with cardiogenic shock requiring multiple inotropes, acute myocardial infarction with indication for urgent revascularization, systolic blood pressure (SBP) ≥160 mmHg (recorded at the time of recruitment) on ≥3 antihypertensive drugs, serum potassium >5.1 mmol/L, estimated glomerular filtration rate (eGFR) <15 mL/min/1.73 m^2^, or recent HF rehospitalization (Figure 1).

### 2.2. Clinical Characteristics

Baseline sociodemographic, clinical, and laboratory data were collected on the day of recruitment and entered into a secure REDCap database hosted at the University of the Witwatersrand. Blood pressure and anthropometric measurements were obtained according to standard procedures; body mass index (BMI) was calculated as weight (kg)/height^2^ (m^2^). All patients underwent 2-dimensional transthoracic echocardiography, including tissue Doppler and speckle tracking, using Vivid^TM^ iq and Vivid E95 (GE Healthcare, Chicago, IL, USA), equipped with 4VC and M5Sc-D transducers, operating at frequencies of 1.5 to 4.0 MHz and 1.4 to 4.6 MHz, respectively. All measurements were performed in accordance with the recommendations of the European and American Society of Echocardiography [12]. Left ventricular ejection fraction (EF) was calculated using the modified Simpson’s method and used to classify heart failure (HF) phenotypes as HFrEF (<40%), HFmrEF (40–49%), and HFpEF (≥50%). Measurements were averaged over three to five cardiac cycles in patients with sinus rhythm and five to ten cycles in those with atrial fibrillation.

Venous blood samples were analyzed at the National Health Laboratory Service. Parameters included serum sodium, potassium, urea, creatinine, NT-proBNP, full blood count, C-reactive protein (CRP), HbA1c, troponin T, and lipid profile. The estimated glomerular filtration rate was calculated using the CKD-EPI equation.

### 2.3. Non-Cardiac Comorbidities and Outcomes

We evaluated 10 NCCs among the patients admitted with acute HF based on their relevance to HF outcomes. These included (1) anaemia/haemoglobin < 12 g/dL (women) and <13 g/dL (men); (2) CKD: eGFR < 60 mL/min/1.73 m^2^; (3) diabetes mellitus: HbA1c ≥ 6.5%, documented diagnosis, or medication use; (4) obesity: BMI ≥ 30 kg/m^2^; (5) dyslipidaemia: Low density lipoprotein-C (LDL-C) ≥ 3.37 mmol/L; (6) chronic obstructive pulmonary disease (COPD); (7) retroviral infection; (8) cancer; (9) thyroid disorders (defined as known hypothyroidism on stable replacement therapy; no cases of hyperthyroidism were observed); and (10) systemic lupus erythematosus. The primary outcomes were all-cause in-hospital mortality and length of hospital stay.

### 2.4. Statistical Analysis

Categorical variables were summarized as frequencies and percentages and compared across comorbidity groups using the Chi-square test or Fisher’s exact test. The distribution of continuous variables was assessed using the Shapiro–Wilk test. Normally distributed variables were reported as mean ± standard deviation and compared using one-way ANOVA. In contrast, non-normally distributed variables were expressed as median (interquartile range) and compared using the Kruskal–Wallis test. Post hoc comparisons were adjusted using the Bonferroni method.

Associations between comorbidity burden and clinical outcomes (in-hospital mortality and length of stay) were examined in two steps. First, univariate analyses were performed to evaluate crude associations. Second, multivariable models were constructed to adjust for potential confounders. Variables with *p* < 0.20 in univariate analysis and clinically relevant covariates (age, sex, systolic blood pressure, heart rate, KCCQ score, and log-transformed NT-proBNP) were included in the final models. Logistic regression was used to model in-hospital mortality and results are presented as adjusted odds ratios (aORs) with 95% confidence intervals. Linear regression was used to assess predictors of length of stay and results are reported as β-coefficients and 95% confidence intervals.

To confirm the robustness of the findings, we performed a sensitivity analysis using propensity score-based inverse probability weighting (IPW). Propensity scores were estimated using logistic regression with key baseline covariates, and the inverse of these scores was used to create balanced weighted groups. Weighted-average treatment effects were then calculated to estimate the impact of high comorbidity burden (≥3 NCCs) on outcomes. A standardized mean difference of <0.1 was considered an acceptable covariate balance.

A two-tailed *p* < 0.05 was considered statistically significant. All analyses were performed using STATA SE version 18.5 (StataCorp LLC, College Station, TX, USA).

## 3. Results

### 3.1. Study Population

Of the 705 patients screened, 406 were included in the final analysis (Figure 1). The most common reasons for exclusion were end-stage renal disease, acute coronary syndrome requiring percutaneous coronary intervention, rehospitalization for HF, and cardiogenic shock. Among the included patients, 63.3% had HFrEF, 15.3% had HFmrEF, and 21.4% had HFpEF. The baseline characteristics of the patients with HF according to HF phenotype is presented in Table 1.

### 3.2. Distribution and Burden of Non-Cardiac Comorbidities

**Figure 2:**
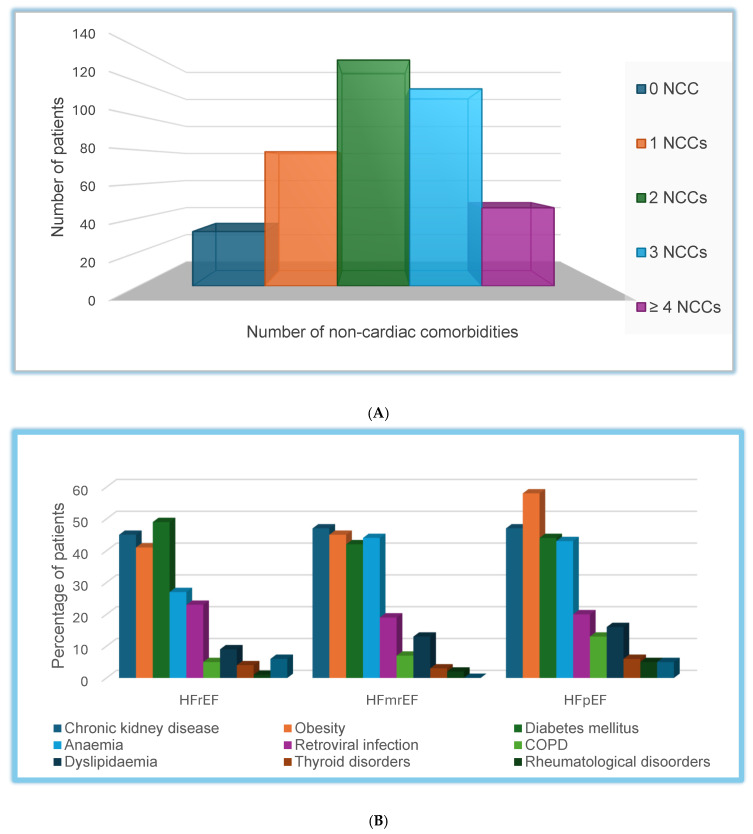
(**A**) Distribution of non-cardiac comorbidity burden. This figure shows the number of non-cardiac comorbidities (NCCs) in the study cohort, grouped into 0, 1, 2, 3, or ≥4 NCCs. NCCs = non-cardiac comorbidities. (**B**) Stacked bar chart of individual non-cardiac comorbidities (NCCs) by HF phenotype. This figure shows the prevalence of individual non-cardiac comorbidities by heart failure phenotype. HF phenotypes were categorized as heart failure with reduced ejection fraction, mildly reduced ejection fraction, and preserved ejection fraction. NCCs include diabetes mellitus, chronic kidney disease, obesity, anaemia, human immunodeficiency virus (HIV) infection, chronic obstructive pulmonary disease, dyslipidaemia, thyroid disorders, systemic lupus erythematosus, and cancer. Each bar represents the proportion of patients with NCC within a phenotype. COPD = Chronic obstructive airway disease; HFmrEF = Heart failure with mildly reduced ejection fraction; HFpEF = Heart failure with preserved ejection fraction; HFrEF = Heart failure with reduced ejection fraction.

Non-cardiac comorbidities were highly prevalent. The most common were diabetes (47%), CKD (46%), obesity (45%), and anaemia (33%). Nearly three-quarters of patients had ≥2 NCCs. The distribution of comorbidities varied by HF phenotype (Table 2, Figure 2B). Obesity and anaemia were significantly more common in HFmrEF/HFpEF than in HFrEF (both *p* < 0.01). Other comorbidities, including CKD, diabetes, human immunodeficiency virus infection, and chronic obstructive pulmonary disease, were evenly distributed across HF phenotypes.

### 3.3. Clinical Profile According to Comorbidity Burden and Medication Use

Patients with ≥3 NCCs had a markedly different clinical profile (Table 1). They were older, had significantly higher BMI and worse renal function (all *p* < 0.01). NT-proBNP levels were numerically higher but not significantly different across categories. Left ventricular systolic function improved across categories of comorbidity burden, with the highest EF observed in patients with ≥4 NCCs (*p* = 0.014). Loop diuretics were used in >95% of patients. Use of mineralocorticoid receptor antagonists decreased with increasing comorbidity burden, while calcium channel blocker use was highest among patients with ≥4 NCCs (*p* = 0.012).

**Table 1:** Baseline characteristics and comorbidities stratified by the number of non-cardiac comorbidities.

Variables	No NCCs (n = 32)	1 NCCs (n = 79)	2 NCCs (n = 133)	3 NCCs (n = 116)	≥4 NCCs (n = 46)	*p*-Value
Age, years *	47.78 ± 16.0	52.41 ± 16.51	53.88 ± 15.79	59.79 ± 15.18	55.11 ± 13.25	**0.004**
Female sex, n (%)	8(25)	29 (60.3)	75 (56.4)	63 (54.3)	32 (69.6)	**<0.001**
NYHA III-IV, n (%)	17 (53)	52 (66)	85 (64)	74 (64)	30 (64)	**<0.001**
BMI, kg/m^2 !^	22.95 (21.6–26.65)	26.70 (24.4–29.80)	28.70 (24.6–34.20)	30.45 (25.55–35.05)	34.95 (31.60–37.90)	**<0.001**
SBP, mmHg ^!^	113 (103.5–129.5)	119 (107–131)	119 (106–130)	119 (105–134)	120 (107–139)	0.597
DBP, mmHg *	75.03 ± 15.34	75.0 ± 12.51	74.0 ± 14.15	75.8 ± 14.59	75.0 ± 13.24	0.561
NT-proBNP, pg/mL ^!^	5007 (2244.5–9663.5)	4528 (2232–11,031)	4605 (1880–9434)	6578.5 (2050.5–14,479)	4421 (2126–10,368)	0.162
Creatinine, μmol/L ^!^	96.5 (76–110.5)	94 (77–120)	92 (79–112)	121.5 (88–154.5)	138 (106–195)	**<0.001**
eGFR, mL/min/1.73 m^2 !^	77.5 (65–88.5)	69 (61–90)	68 (53–87)	49.5 (36–63.5)	41.5 (30–48)	**<0.001**
CRP, mg/L ^!^	21.5 (19–72)	30 (15–69)	35 (16–69)	33 (16–58)	37 (21.5–88)	0.851
LVEF (%)	25.5 (19.5–36)	28 (17–42)	32 (22–48)	33.5 (22–48)	39 (25–57)	**0.014**
LAD mm *	45.19 ± 8.55	47.10 ± 7.46	47.43 ± 7.07	44.93 ± 7.42	45.59 ± 8.97	0.073
LVIDd, mm *	57.16 ± 11.09	56.44 ± 10.83	54.70 ± 11.23	54.16 ± 9.45	50.89 ± 10.05	**0.029**
PWTd, mm *	11.69 ± 3.16	11.63 ± 2.37	12 ± 2.99	12.03 ± 2.92	12.61 ± 3.50	0.462
LVMI, g/m^2 !^	165.5 (133.5–221)	147 (123–178)	157 (130–193)	151 (126.5–191)	123 (99–164)	**0.007**
RVFAC, % ^!^	30.5 (21.5–39)	31 (24–43.5)	31 (21–42)	29 (22–43)	37 (26–44)	0.312
E/A ^!^	2.03 (0.93–2.55)	1.82 (1.18–2.79)	1.68 (1.09–2.48)	1.45 (0.85–2.42)	1.27 (0.87–1.95)	0.118
E/e’ lateral ^!^	14.7 (8.14–17.41)	12.9 (8.9–17.4)	12 (8.6–17.2)	12.5 (8.27–18.35)	13.47 (9.5–20.2)	0.713
GLS, % ^!^	−6.2 (−7.3, −4.8)	−5.3 (−9.9, −3.9)	−6.5 (−9.5, −4.1)	−5.6 (−8.9, −4.0)	−6.4 (−8.5, −3.9)	0.819
Loop diuretic, n (%)	31 (97)	75 (95)	128 (96)	113 (97)	44 (96)	0.924
ACEIs/ARBs, n (%)	30 (94)	67 (85)	113 (85)	106 (91)	37 (80)	0.234
MRA, n (%)	28 (88)	66 (84)	112 (84)	91 (78)	33 (72)	0.268
CCBs, n (%)	5 (16)	8 (10)	9 (7)	17 (15)	12 (26)	**0.012**
B-blockers, n (%)	30 (94)	67 (85)	118 (89)	95 (82)	40 (87)	0.329
SGLT2 inhibitors, n (%)	5 (16)	7 (9)	15 (11)	11 (9)	6 (13)	0.816
Length of stay, days ^!^	8 (5–12)	8 (5–13)	8 (6–14)	10 (6.5–15)	8 (6–150)	0.229
In-hospital mortality, n (%)	0 (0)	3 (4)	4 (3)	4 (3)	3 (7)	0.318

* = Mean ± SD; ! = Median (IQR) Abbreviations: A wave = peak late diastolic transmitral flow velocity; ACEIs = angiotensin-converting enzyme Inhibitors; ARBs = angiotensin receptor blockers; BMI = body mass index; CCBs = calcium channel blockers; CRP = C-reactive protein; DBP = diastolic blood pressure; E wave = peak early diastolic transmitral flow velocity; e’ = peak early diastolic mitral annular tissue velocity; GLS = global longitudinal strain; IQR = interquartile range; LVIDd = left ventricular internal dimension end-diastole. Bold values indicate statistical significance at *p* < 0.05.

**Table 2:** Prevalence of Non-Cardiac Comorbidities by HF Phenotype.

Variables	HFrEF (n = 257)	HFmrEF (n = 62)	HFpEF (n = 87)	*p*-Value
Chronic kidney disease, n (%)	115 (45)	29 (47)	41 (47)	0.909
Obesity, n (%)	105 (40.9)	28 (45.2)	50 (61)	**<0.001**
Diabetes mellitus, n (%)	126 (49)	26 (42)	38 (44)	0.486
Anaemia, n (%)	69 (27)	27 (44)	37 (43)	**0.004**
Retroviral infection, n (%)	58 (23)	12 (19)	17 (20)	0.861
COPD, n (%)	12 (4.7)	4 (6.5)	10 (11.5)	0.080
Dyslipidaemia, n (%)	22 (8.7)	8 (12.90	14 (16.1)	0.122
Thyroid disorders, n (%)	10 (3.9)	2 (3.23)	5 (5.8)	0.695
Systemic lupus erythematosus, n (%)	3 (1.2)	1 (1.6)	4 (4.6)	0.094
Cancers, n (%)	14 (5.5)	0 (0)	4 (4.6)	0.173

COPD = Chronic obstructive airway disease; HFmrEF = Heart failure with mildly reduced ejection fraction; HFpEF = Heart failure with preserved ejection fraction; HFrEF = Heart failure with reduced ejection fraction. Bold values indicate statistical significance at *p* < 0.05.

### 3.4. Length of Stay and In-Hospital Mortality

**Figure 3:**
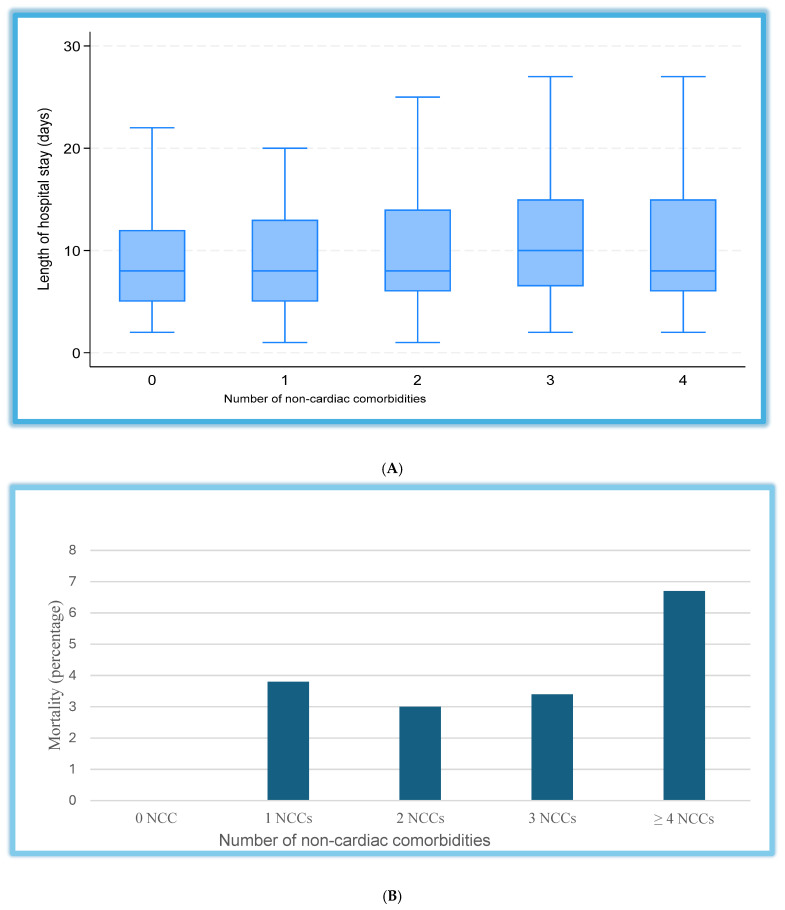
(**A**) Boxplot of length of stay by number of non-cardiac comorbidities: This figure shows the distribution of hospital length of stay by non-cardiac comorbidity (NCC) burden. Patients were grouped according to the number of NCCs: 0, 1, 2, 3, and ≥4. The box represents the interquartile range (IQR), the horizontal line indicates the median, and the whiskers denote the range excluding outliers. (**B**) In-hospital mortality across non-cardiac comorbidity (NCC) groups. Absolute event numbers were low (n = 0–3), and the figure is intended to illustrate trends rather than indicate statistically significant differences. Percentages indicate the mortality in each NCC category. NCCs = non-cardiac comorbidities.

The overall median hospital stay was 8 days (IQR: 5–12), and patients with a higher non-cardiac comorbidity burden tended to have a modestly longer duration of admission (Figure 3). In univariate analysis, only thyroid disorders and heart rate were significantly associated with the length of hospital stay. In multivariable linear regression analysis, the overall model predicting length of hospital stay was statistically significant (F(17,388) = 2.31, *p* < 0.001; R^2^ = 0.109). Among individual predictors, the use of renin–angiotensin system inhibitors (RASi) and the presence of thyroid disorders were independently associated with shorter hospital stay (β = −1.84 days, 95% CI: −3.78, −0.12, *p* = 0.049; β = −4.10 days, 95% CI: −7.75, −0.45, *p* = 0.028, respectively). In contrast, a higher heart rate at admission was associated with prolonged hospitalization (β = +0.05 days per beat per minute, 95% CI: 0.006 to 0.092, *p* = 0.025). Systolic blood pressure showed a borderline association with shorter stay (β = −0.04 days, 95% CI: −0.083–0.004, *p* = 0.077). Age, sex, diabetes, chronic kidney disease, and higher comorbidity burden were not significantly associated with hospital duration after adjustment (Supplementary Table S1).

### 3.5. Univariate, Multivariate, and Sensitivity Analyses

After multivariable adjustment, a higher heart rate was associated with longer hospital stay, while use of RAS inhibitors and the presence of thyroid disorders (well-treated patients) were associated with shorter hospital stay (all *p* < 0.05). In-hospital mortality was low overall (3.4%) and increased with the number of comorbidities, but differences between groups were not significant (*p* = 0.318). In multivariable analysis, only KCCQ score and NT-proBNP independently predicted mortality. Each one-point decrease in KCCQ score was associated with 0.9% higher odds of death (OR 1.009, 95% CI 1.003–1.015, *p* = 0.005), while each 1-unit increase in log-transformed NT-proBNP, corresponding approximately to a 2.7-fold increase in absolute NT-proBNP, was associated with an 85% higher odds of death (aOR 1.85; 95% CI: 1.07–3.50; *p* = 0.026). Other covariates, including age, sex, SBP, diabetes mellitus, CKD, higher non-cardiac comorbidity burden, and RASi use, were not significantly associated with in-hospital mortality (all *p* > 0.05) (Supplementary Table S2). The sensitivity analyses done using inverse probability weighting produced similar findings. Higher comorbidity burden showed a trend toward longer hospital stay but was not associated with mortality (aOR = 2.9, 95% CI: 0.70–11.76, *p* = 0.142; IPW logit with an ATE = +0.016, 95% CI: −0.03–0.06, *p* = 0.482). The direction and magnitude of the estimates were consistent across analytical models, supporting the robustness of the results.

## 4. Discussion

In this single-center observational study of 406 patients hospitalized with acute HF in Johannesburg, South Africa, we found a high burden of NCCs across all HF phenotypes, with more than two-thirds of patients presenting with ≥2 comorbid conditions. CKD, obesity, diabetes mellitus, and anaemia were the most prevalent NCCs, reflecting the emerging cardiometabolic profile of HF in sub-Saharan Africa. Lower KCCQ scores and higher NT-proBNP levels were independently associated with increased in-hospital mortality, whereas a higher admission heart rate was associated with longer hospitalization. In contrast, the use of RASi and the presence of thyroid disorders were associated with a shorter length of stay.

The clinical course of acute HF is shaped not only by cardiac dysfunction but also by noncardiac comorbidities, which frequently drive hemodynamic instability, treatment limitations, and poor outcomes. These conditions are often under-recognized; however, they contribute to longer hospital stays, higher mortality, and reduced uptake of GDMT [2,4,5,6,7]. Our findings are consistent with those of previous large registries which have demonstrated a similarly high comorbidity burden in acute HF [4,6,13]. For example, in the ESC-HFA Long-Term Registry, 80% of hospitalized patients with HF had at least one NCC, and multimorbidity independently predicted both short- and long-term mortality. Comparable estimates have been reported in Asian and European cohorts, where 80–85% of patients presented with ≥1 NCC [14,15]. Although the comorbidity profile in SSA differs from these settings, reflecting a dual burden of infectious and metabolic diseases, our lack of an observed mortality association contrasts with the Medicare and ESC-HFA data [4,5]. This is most likely attributable to the small number of deaths in our cohort, exclusion of high-risk patients, and limited statistical power, rather than regional differences alone. The relatively high prevalence of HIV in our cohort further illustrates this distinct comorbidity pattern. It aligns with INTER-CHF and other studies, which also reported higher rates of infectious disease and anaemia in SSA than in other regions [16,17,18].

Patients with higher NCC burden in our cohort were older, more often female, and had higher BMI and worse renal function. CKD, anaemia, obesity, and diabetes were particularly prominent among patients with HFpEF and HFmrEF. This comorbidity clustering has been reported in other HF cohorts and supports mechanistic models where systemic inflammation, metabolic dysfunction, and microvascular disease contribute to myocardial fibrosis and diastolic dysfunction [19,20,21,22,23]. Importantly, patients with ≥4 NCCs in our study were significantly less likely to receive GDMT, especially mineralocorticoid receptor antagonists. This finding mirrors real-world data showing that multimorbidity is associated with lower GDMT prescription and a reduced likelihood of therapy intensification in patients with ≥4 comorbidities [24,25,26]. Potential explanations include renal impairment, risk of hyperkalaemia, and greater clinical complexity. The higher use of calcium channel blockers in those with multimorbidity likely reflects pragmatic treatment decisions focused on blood pressure and symptom control rather than neurohormonal modulation. This is consistent with pooled trial cohorts of HFpEF/HFmrEF, where up to 35% of patients received CCBs [27].

Interestingly, patients with thyroid disorders in our cohort had a shorter length of stay. This contrasts with prior studies, which have reported that hypothyroidism and subclinical hyperthyroidism are associated with worse outcomes, including higher morbidity and mortality in HF [28,29,30]. Several factors may explain this discrepancy. First, most patients were already on stable thyroid replacement therapy rather than presenting with overt thyrotoxicosis or untreated hypothyroidism, which may mitigate clinical deterioration. Restoration of euthyroidism has been shown to improve haemodynamics and symptoms in HF, potentially influencing short-term outcomes [31,32]. Second, the number of patients with thyroid disorders was small, and the effect estimate should therefore be interpreted cautiously. Furthermore, acute thyroid states requiring specialist intervention were excluded, which likely selected for a less severe group. Thus, the observed shorter length of stay is more likely attributable to selection effects and residual confounding than to an actual protective effect of thyroid disease. While these findings reinforce the clinical relevance of identifying and managing thyroid dysfunction in HF, they remain hypothesis-generating and require confirmation in larger cohorts with more granular phenotyping.

Although crude differences in in-hospital mortality and length of stay did not reach statistical significance, both outcomes exhibited a stepwise worsening with increasing NCC burden. This pattern aligns with global evidence demonstrating that multimorbidity independently predicts poorer short-term outcomes [13,33,34,35]. In SSA, limited access to advanced therapies, specialist care, and fragmented chronic disease management may further amplify the impact of a high NCC burden on hospital resources and patient outcomes [16,36]. Lower KCCQ scores were associated with higher in-hospital mortality, highlighting the prognostic value of patient-reported health status in acute HF. This is consistent with prior studies showing that reduced KCCQ scores predict increased rehospitalization and mortality [37]. Similarly, higher resting heart rate independently correlated with longer hospitalization, supporting previous findings that tachycardia contributes to greater hemodynamic stress, impaired ventricular efficiency, and adverse short-term outcomes in acute HF [38]. Collectively, these results underscore the importance of integrating both objective clinical measures and patient-reported outcomes when evaluating disease severity and short-term prognosis in acute HF.

Sensitivity analyses using multivariable adjustment and propensity score weighting demonstrated consistent trends, reinforcing the additive impact of multimorbidity on healthcare utilization. Although differences in in-hospital outcomes of individual NCC did not reach statistical significance, likely due to limited power, exclusion of critically ill patients, and low event rates, the observed patterns align with those in larger international cohorts, where a higher comorbidity burden prolongs hospital stays and increases post-discharge readmissions [5,33,39,40,41,42,43]. In our cohort, exclusion of patients with cardiogenic shock, ongoing ACS, or advanced renal failure resulted in a lower-acuity HF population, in which immediate drivers of mortality and length of stay are likely related to presentation severity and hemodynamic status rather than chronic comorbidities. Consequently, the prognostic effect of NCCs may appear attenuated in this context, but this does not diminish their overall clinical relevance. These findings underscore that, beyond comorbidity burden, functional status, neurohormonal activity, and biomarkers of myocardial stress are key determinants of short-term outcomes in acute HF. Integrating multimorbidity assessment and inflammatory profiling into HF care pathways may improve risk stratification, support personalized management, and inform the development of phenotype-specific HF strategies tailored to the African context.

## 5. Limitations

This study has several limitations. First, as a single-center study conducted at a public tertiary hospital, the findings may not fully represent the comorbidity burden in rural or community settings across SSA, where healthcare access and disease patterns differ. Second, the relatively low in-hospital mortality may have limited statistical power to detect meaningful differences across NCC burden categories. Although we initially explored multivariable analysis of mortality, the findings should be interpreted as exploratory given the low event rate. Third, the exclusion of patients with cardiogenic shock requiring multiple inotropes, acute coronary syndromes requiring revascularization, and end-stage renal disease may have sanitized the cohort by selecting a lower-risk population. This likely contributed to the low event rates and may have attenuated the observed associations between comorbidities and clinical outcomes. In addition, HIV status was captured as a binary variable without granular data on ART adherence or viral suppression, which may have limited our ability to evaluate the prognostic impact of controlled versus uncontrolled HIV infection. Fourth, inclusion of diverse HF aetiologies may have introduced heterogeneity, potentially affecting observed associations between NCCs and HF phenotypes. Finally, as an observational study, causal inference is limited despite adjustment and sensitivity analyses; these findings should therefore be considered hypothesis-generating. Future studies should include consecutive acute HF patients with longitudinal follow-up to better evaluate causal and prognostic relationships between NCCs and outcomes across SSA populations.

## 6. Conclusions

In this South African cohort of adults hospitalized with acute HF, most patients (68%) had ≥2 noncardiac comorbidities, with CKD, obesity, diabetes, and anaemia being the most common. A higher resting heart rate predicted longer hospitalization, whereas the use of RASi was associated with a shorter stay. Additionally, lower KCCQ scores and higher log-transformed NT-proBNP levels were associated with increased in-hospital mortality. These findings underscore the importance of systematic comorbidity assessment and integration into phenotype-specific care pathways. Such an approach may help improve risk stratification, guide therapy selection, and support the development of evidence-based HF strategies adapted to the specific needs of SSA populations.

## Data Availability

All data are available on request from the corresponding author.

## Supplementary Materials

The following supporting information can be downloaded at https://www.mdpi.com/article/10.3390/jcm15062202/s1, Table S1: Univariate and Multivariate Linear Regression Analysis of Predictors of Length of Hospital Stay (days) in Patients with Acute Heart Failure and Non-Cardiac Comorbidities. Table S2: Univariate and multivariate analysis of In-Hospital Mortality in Acute Heart Failure with Non-Cardiac Comorbidities. Table S3: Sensitivity analyses evaluating the association between comorbidity burden and in-hospital outcomes.
