# Supplementary material for: Non-Cardiac Comorbidities in Acute Heart Failure: Phenotype-Specific Insights from Sub-Saharan Africa
Source: J Clin Med. 2026 Mar 13;15(6):2202. doi: 10.3390/jcm15062202 (PMC13027385; doi:10.3390/jcm15062202)
Supplement: Supplementary file 1 [file jcm-15-02202-s001.zip › jcm-3986096-supplementary.pdf]

Supplementary File

Supplementary Table S1. Univariate and Multivariate Linear Regression Analysis of Predictors of Length of Hospital Stay (days) in Patients with Acute Heart Failure and Non-Cardiac Comorbidities.

**Variable**	**Unadjusted**		**Multivariate analysis**	**p-value**
	β Coefficient (95% CI)	p-value	aβ Coefficient (95% CI)	
**Age (per year)**	0.0003 (-0.04-0.05)	0.990		
**Female sex**	0.06 (-1.41-1.53)	0.935		
**SBP, mmHg**	-0.03 (-0.09-0.13)	0.145		
**Heart rate (per bpm)**	0.06 (0.01-0.10)	0.009	0.05 (0.006-0.092)	**0.025**
**KCCQ**	-0.002 (-0.009-0.004)	0.485		
**SII**	0.0005 (-0.0001-0.001)	0.130		
**Hypertension**	-1.19 (-2.82-0.44)	0.153		
**Anaemia**	0.97 (-0.59-2.54)	0.223		
**Diabetes mellitus**	1.06 (-41-2.53)	0.157		
**CKD**	1.27 (-2.00-2.75)	0.090		
**Obesity**	0.15 (-1.33-1.63)	0.846		
**Malignancy**	2.75 (-0.82-6.31)	0.131		
**Dyslipidaemia**	-1.46 (-3.82-0.91)	0.227		
**HIV infection**	2.02 (0.25-3.81)	0.026		
**COPD**	-1.49 (-4.39-1.41)	0.312		
**Thyroid disorders**	-3.74 (-7.40,-0.09)	0.045	-4.10 (-7.75, -0.45)	**0.028**
**Rheumatological disorders**	-0.96 (-6.26-4.33)	0.720		
**High burden***	1.52 (-0.03-3.02)	0.046		
**NT-proBNP (per unit)**	0.0001 (0.00005-0.0002)	0.002		
**NYHA IV**	3.37 (-0.03-6.76)	0.052		
**Use of RASi**	-1.67 (-3.48-0.14)	0.071	-1.84 (-3.78, -012)	**0.049**
**LVMI**	-0.04 (-0.18-0.01)	0.567		
**EF**	-0.006 (-0.07-0.06)	0.859		
**LAD**	0.05 (-0.05-0.14)	**0.337**		
**GLS**	0.16 (-0.05-0.37)	0.132		

Abbreviations: a = adjusted; β = regression coefficient; CI = confidence interval; CKD = chronic kidney disease; COPD = chronic obstructive pulmonary airway disease; EF = ejection fraction; GLS = global longitudinal strain; HIV = Human immunodeficiency virus; KCCQ = Kansas City Cardiomyopathy Questionnaire; LAD = left atrial dimension; LVMI = left ventricular mass index; NCC = non-cardiac comorbidity; NT-proBNP = N-terminal pro-B-type natriuretic peptide; NYHA = New York Heart Association; RASi = renin–angiotensin system inhibitors; SBP = systolic blood pressure; SII = systemic inflammatory index; *High burden ≥3 NCCs; Bold values indicate statistical significance at $p < 0.05$.

Supplementary Table S2. Univariate and multivariate analysis of In-Hospital Mortality in Acute Heart Failure with Non-Cardiac Comorbidities.

Variable	Univariate analysis		Multivariate analysis	p-value
	OR (95% CI)	p-value	aOR (95% CI)	
**Age (per year)**	1.02 (0.99-1.05)	0.265		
**Sex (female)**	0.52 (0.17-1.59)	0.252		
**SBP, mmHg**	0.97 (0.94-1.00)	0.073		
**Heart rate (bpm)**	1.00 (0.97-1.04)	0.760		
**KCCQ**	1.01 (1.0008-1.01)	0.024	1.009 (1.003-1.015)	**0.005**
**SII**	1.0005(1.0002-1.0006)	0.000		
**Hypertension**	0.69 (0.23-2.11)	0.520		
**Anaemia**	1.56 (0.53-4.61)	0.416		
**Diabetes mellitus**	2.10 (0.69-6.37)	0.191		
**CKD**	6.24 (0.60-64.62)	0.125		
**Obesity**	0.92 (0.32-2.70)	0.880		
**Dyslipidaemia**	0.62 (0.08-4.89)	0.654		
**HIV**	0.60 (0.13-2.74)	0.512		
**Thyroid disorders**	Not selected			
**Malignancy**	Not selected			
**COPD**	1.04 (0.13-8.25)	0.970		
**High burden***	1.53 (0.53-4.44)	0.435		
**NT-proBNP (per unit)**	1.00006 (1.00001-1.0001)	0.015	1.00007 (1.00002-1.00013)	**0.014**
**NYHA IV**	0.54 (0.15-1.97)	0.349		
**Use of RASi**	0.45 (0.15-1.39)	0.168		
**LVMI**	1.00 (0.99-1.01)	0.975		
**EF**	0.99 (0.95-1.02)	0.411		
**LAD**	0.97 (0.91-1.04)	0.440		
**GLS**	0.99 (0.86-1.15)	0.934		

Abbreviations: a = adjusted; CI = confidence interval; CKD = chronic kidney disease; COPD = chronic obstructive pulmonary airway disease; EF = ejection fraction; GLS = global longitudinal strain; HIV = Human immunodeficiency virus; KCCQ = Kansas City Cardiomyopathy Questionnaire; LAD = left atrial dimension; LVMI = left ventricular mass index; NCC = non-cardiac comorbidity; NT-proBNP = N-terminal pro-B-type natriuretic peptide; NYHA = New York Heart Association; OR = Odds ratio; RASi = renin–angiotensin system inhibitors; SBP = systolic blood pressure; SII = systemic inflammatory index. *High burden ≥3 NCCs; Bold values indicate statistical significance at $p < 0.05$.

Supplementary Table S3. Sensitivity analyses evaluating the association between comorbidity burden and in-hospital outcomes.

Model / Method	**Outcome**	**Effect Estimate (95% CI)**	**p-value**	**Interpretation**
**Multivariable logistic regression**	In-hospital mortality	aOR = 2.9 (0.70-11.76)	0.142	No significant association
**Multivariable linear regression**	Length of hospital stay (days)	β = +1.4 (-0.09-2.91)	0.066	Trend toward longer stay
**IPW model (logit)**	In-hospital mortality	ATE = +0.016 (-0.03-0.06)	0.482	No significant association
**IPW model (weighted mean)**	Length of hospital stay (days)	ATE = +1.4 (-0.09-2.91)	0.066	Trend toward longer stay

aOR = adjusted odds ratio; ATE = average treatment effect; β = regression coefficient; IPW = inverse probability weighting
